# Masked face is looking at me: Face mask increases the feeling of being looked at during the COVID-19 pandemic

**DOI:** 10.3389/fnins.2022.1056793

**Published:** 2022-11-24

**Authors:** Jiakun Liu, Jiajia Yang, Lihui Huang, Li Zhou, Jinxi Xie, Zhonghua Hu

**Affiliations:** ^1^Institute of Brain and Psychological Sciences, Sichuan Normal University, Chengdu, China; ^2^Jinhua Middle School, Suining, China; ^3^Research Center of Brain and Cognitive Neuroscience, Liaoning Normal University, Dalian, China

**Keywords:** face mask, gaze direction, mask type, the cone of direct gaze (CoDG), COVID-19

## Abstract

**Background:**

As the COVID-19 global pandemic unfolded, governments recommended wearing face masks as a protective measure. Recent studies have found that a face mask influences perception; but how it affects social perception, especially the judgment of being looked at, is still unknown. This study investigated how wearing a mask influences the judgment of gaze direction by conducting a cone of direct gaze (CoDG) task.

**Methods:**

In Experiment 1, three types of masked faces were considered to investigate whether the effect of masks on CoDG is modulated by mask types. Experiment 2 was to further validate the results of Experiment 1 by adding a learning phase to help participants better distinguish N95 and surgical masks. Furthermore, to investigate whether the effect of masks derives from its social significance, a face with only the eye-region (a mouth-cut face) was used as the stimuli in Experiment 3.

**Results:**

The results of Experiment 1 found that wearing masks widens the CoDG, irrespective of the mask type. Experiment 2 replicated the results of Experiment 1. Experiment 3 found that the CoDG of N95-masked faces was wider than the mouth-cut and non-masked faces, while no significant difference existed between the CoDG of mouth-cut and non-masked faces, illustrating that the influence of wearing masks on CoDG was due to high-level social significance rather than low-level facial feature information.

**Conclusion:**

The results show that face mask increases the feeling of being looked at during the COVID-19 Pandemic. The present findings are of significance for understanding the impact of wearing masks on human social cognition in the context of COVID-19.

## Introduction

In 2019, a new infectious disease caused by the SARS-CoV-2 began spreading across the world. The World Health Organization (WHO) named it the coronavirus disease (COVID-19). Against the backdrop of the COVID-19 pandemic, wearing a mask has become a common phenomenon, since it is an important tool to effectively block the spread of the droplets that cause this disease. However, wearing a mask may have negative effects as well. Some studies recruited normal adults as participants and have found that masks provide a somewhat obscuring effect on the face, as they cover the nose and mouth areas of the wearer’s face, which is believed to affect the recognition of facial emotions (Carbon, 2020; Noyes et al., 2021) and influences one’s judgment of the wearer’s trustworthiness (Cartaud et al., 2020).

### Cone of direct gaze is an index of the feeling of being looked at

The eye region, a facial area that is not covered by masks, shows the direction of eye gaze and can provide important information such as attentional location, behavioral intention, and emotional state (Emery, 2000; Mareschal et al., 2013b; 2014). Perceiving that a person is looking at us (Bateson et al., 2006; Carbon and Hesslinger, 2011; Hamilton, 2016), or looking away from us (Hietanen et al., 2008; Colombatto et al., 2020), has different effects on our behavior and perception. Therefore, an accurate perception of the gaze direction of one’s eyes is crucial to social interaction. However, previous studies have found that the perception of one’s gaze direction may not be very accurate. Humans prefer to judge others by looking at them (Mareschal et al., 2013a). To measure this tendency, Jun et al. (2013) conducted a study where participants were instructed to judge whether they perceived the gaze on faces looking in various directions to be looking at them. This index is commonly known as the cone of direct gaze (CoDG) (Gamer and Hecht, 2007; Jun et al., 2013). The wider the CoDG, the more the participant perceived the face as “looking at me.”

### Factors influencing cone of direct gaze

Although CoDG is a relatively stable indicator for individuals (Lobmaier et al., 2021), it is influenced by a variety of factors. Firstly, facial information, such as head orientation (Gamer and Hecht, 2007), facial attractiveness (Kloth et al., 2011), facial expressions (Ewbank et al., 2009; Gillian et al., 2012), and so on, can influence CoDG. Secondly, CoDG is also affected by individual differences among the participants. People with autistic traits are less likely to judge being looked at, which means their CoDG is narrower (Matsuyoshi et al., 2014). Anxious individuals (Harbort et al., 2013) and schizophrenic patients (Wastler and Lenzenweger, 2018) are more inclined to judge someone looking at them as compared to others, meaning that their CoDG is wider. In addition, a recent study found that emotional situations may also affect CoDG (Rimmele and Lobmaier, 2012; Lyyra et al., 2017; Syrjämäki et al., 2017). Specifically, external pressure affects perceptual judgments of being looked at; being socially excluded increases one’s judgment of being looked at if there is a possibility of re-interaction (Lyyra et al., 2017) while it deceases if there is no interaction (Syrjämäki et al., 2017).

### The current study

To sum up, CoDG is affected by emotional situations and wearing a mask can create a safe or a threatening situation, especially in the context of the COVID-19 outbreak. Thus, a CoDG task was conducted to examine how wearing a mask impacts the judgment of gaze direction. Different types of masks create different levels of perception of danger or safety (Chu et al., 2020). For example, N95 masks provide a higher level of protection from viruses than regular surgical masks. Unlike a surgical mask, an N95 one may be more likely to convey the message that the virus will not be spread by the individual wearing it. At the same time, wearing N95 masks may also mean that the surrounding environment is dangerous and the likelihood of infection is high. Therefore, three types of masked faces (non-masked faces, surgical-masked faces, and N95-masked faces) were considered in our study to further explore whether the effect of masks on CoDG is modulated by the type of mask being used. In addition, to investigate whether the impact of masks on CoDG was related to COVID-19 or a person’s individual characteristics, we asked the participants to complete a three-item questionnaire on COVID-19, Social Interaction Anxiety Scale (SIAS) (Mattick and Clarke, 1998), and Self-Rating Depression Scale (SDS) (Zung et al., 1965), after the CoDG task.

Given that previous research has already shown that wearing a mask can reduce social distance and increase willingness to socialize in the context of the COVID-19 pandemic (Cartaud et al., 2020), we predicted that participants will over-report their sensation of being looked at. Namely, the CoDG of the masked faces will be wider than that of the unmasked faces. In addition, previous studies have found that while trustworthiness or threat can widen the CoDG, wearing a mask may enhance the wearer’s sense of trustworthiness or threat. Thus, we predict that N95-masked faces may produce a wider CoDG than a face wearing a regular surgical mask.

## Experiment 1

### Methods

#### Participants

An *a priori* power analysis (G*Power 3; Faul et al., 2007) with a medium effect size of 0.25, a 1-β power of 0.80, and an alpha of 0.05 found that the required number of participants in the study should be 28. In addition, the sample size in similar studies has been restricted to 20–40 participants (Ewbank et al., 2009; Pantelis and Kennedy, 2016; Lyyra et al., 2017; Awad et al., 2019). Thus, we expected to test 30 participants. When 30 participants were tested, we found that a large number of participants would have to be excluded due to fitting failure (see section “Data analyses” for details). Finally, we expanded the number of participants to 40 (14 males, 26 females), aged 18–23 years (*M* = 19.6 *SD* = 1.08). All the participants had normal or corrected-to-normal vision and self-reported absence of mental illness. The study was approved by the ethics committee of the Institute of Brain and Psychological Sciences, Sichuan Normal University [SCNU-210520]. All the participants provided their written informed consent to take part in this study and received monetary compensation for their participation (see Supplementary Appendix 1). This study was not pre-registered.

#### Stimuli, materials, and apparatus

The colored, full-face images of six Chinese adult models (3 males, 3 females) were taken using a camera. The models were asked to keep their faces neutral and change their gaze direction continuously at 11 gaze deflection angles (2°, 4°, 6°, 8°, 10° each to the left and right, and 0°) without making any other movement. To exclude the influence of color on the experimental results, we set the color of both masks as white. The models were required to repeat the procedure in their masked condition, wearing a surgical mask or an N95 mask.

**FIGURE 1:**
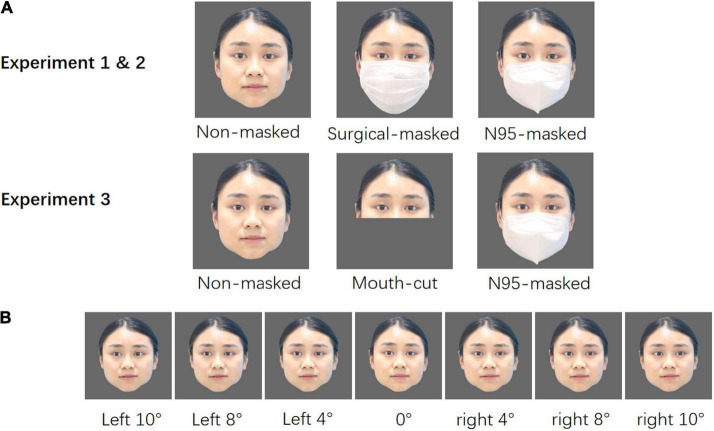
**(A)** Sample of a female model displaying the three face types used in Experiments 1, 2 and 3. **(B)** Sample of a female model displaying the seven gaze directions: 4°, 8°, 10° each to the left and right, and 0°. Written informed consent was obtained from the model for the publication of her images in this article.

All the photographs were edited using Photoshop CS6 (596 × 596 pixels), keeping all the faces consistent in terms of brightness and contrast, maintaining a gray background (see Figure 1).

The experiment was conducted on a 24-inch (1,920 by 1,080 pixels; 60 Hz refresh rate) LCD monitor. Stimulus presentations and recordings of the behavioral measures were controlled by E-prime 2.0.

#### Task and procedure

A 3 (face type: non-masked faces, surgical-masked faces, N95-masked faces) × 11 (gaze direction: 2°, 4°, 6°, 8°, 10° each to the left and right, and 0°) within-subjects design was used. The participants were seated comfortably in a dimly lit room where they received written instructions for the CoDG task. They sat at a distance of ∼60 cm from a LCD monitor. Lighting conditions were kept constant for all the participants and the screen position was manually adapted so that the eyes of the avatars were vertically aligned with the eyes of the participants.

Each trial began with a fixation cross presented on the screen for 1,000 ms. Next, a face with or without a mask was presented, which remained on the screen until the participant’s response. This was followed by a 500 ms blank screen, after which the next trial began. The participants were required to identify the gaze direction by pressing the keyboard (with 1 meaning that “the face is watching my left,” 2 referring to “the face is watching me,” and 3 meaning that “the face is watching my right”). Although each model had faces wearing three types of masks, each participant observed the faces of each model wearing only one type of mask. It was ensured that the face type matched the model for each participant. The binding of a mask to a model’s identity was randomized and balanced among the participants. The presentation sequences of the faces were also random. Each participant completed a total of 594 trials (18 trials per face type × gaze direction). The whole procedure lasted 30 min. After the CoDG task, the participants were instructed to fill three self-assessment questionnaires pertaining to COVID-19, SIAS, and SDS.

#### Data analyses

**FIGURE 2:**
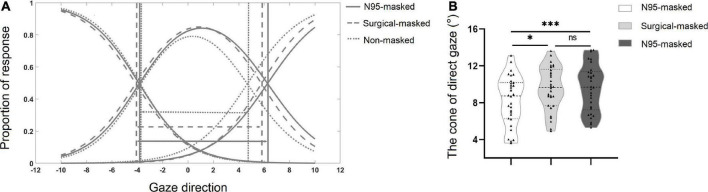
**(A)** Plot showing the mean fitted logistic functions for *left*, *right*, and *direct* responses. The solid lines indicate the N95-masked face condition, the dashed lines indicate the surgical-masked face condition, while the dotted lines indicate the non-masked faces condition. The arrows indicate the width of the cone of direct gaze. **(B)** The mean width of the cone for non-masked face, surgical-masked face, and N95-masked face conditions. ▲ Indicated data for each participant. *p < 0.05, ^***^p < 0.001, ns *p* > 0.05.

The cones of direct gaze were measured using conventional methods. The data were separated into different mask conditions, resulting in three data sets (non-masked faces, surgical-masked faces, N95-masked faces) (see Figure 2A). For each condition, logistic functions were fitted to the proportion of *left* and *right* responses. A function for *direct* responses was calculated by subtracting the sum of the *left* and *right* responses from 1. These three functions were fitted as an ensemble using the Nelder-Mead simplex method (Nelder and Mead, 1965), implemented using the Matlab’s fminsearch function to minimize the residual variance. The cone of direct gaze was calculated as the distance (in degrees of gaze deviation) between the points of intersection (termed categorical boundaries) of the two averted curves with the *direct* curve: one where the *left* and *direct* responses crossed each other and the other where the *direct* and *right* responses intersected.

Ten participants were excluded finally because their response curves were too broad to allow confident estimations of the width of their CoDG, remaining 30 participants’ data (10 males, 20 females) for the statistical analysis.

### Results and discussion

#### Cone of direct gaze

A one-way repeated-measures ANOVA test showed a significant main effect of the face type on the CoDG, [*F*(2, 58) = 6.20, *p* = 0.004, η*_*p*_^2^* = 0.176] (see Table 1). A Bonferroni *post-hoc* test found that the CoDG for N95-masked faces and surgical-masked faces were higher as compared to the non-masked faces, *p* = 0.001, *p* = 0.036. However, there was no significant difference between the CoDG for surgical-masked faces and N95-masked faces, *p* > 0.05 (see Figure 2B).

**TABLE 1:** The CoDG (mean ± SD) on Experiments 1, 2 and 3.

	Experiment 1	Experiment 2	Experiment 3
Non-masked face	8.26 ± 2.67	8.06 ± 2.70	8.57 ± 2.39
Surgical-masked face	9.52 ± 2.36	8.86 ± 2.69	/
N95-masked face	9.60 ± 2.57	8.84 ± 2.65	9.61 ± 2.20
Mouth-cut face	/	/	8.98 ± 2.19

#### Correlations between cone of direct gaze and questionnaires

To assess the relationship between the CoDG and the participants’ knowledge about masks as well as between the CoDG and traits of anxiety and depression, we calculated Pearson correlations between the CoDG of a particular face type and the participants’ responses to questions about face masks, SDS, and SIAS. Bonferroni correction revealed there was no significant correlation, *p*s > 0.05 (see Supplementary Appendix 2).

The results of Experiment 1 showed that wearing masks widened one’s CoDG significantly. Individuals were more likely to judge that a face wearing a mask was looking at them. Contrary to our expectations, there was no significant difference in the CoDG of N95-masked faces and surgical-masked faces, suggesting that the mask type does not affect the CoDG.

## Experiment 2

No effect of different types of face masks on CoDG was found in Experiment 1. One possible explanation is that individuals are not able to distinguish between surgical masks and N95 masks in terms of function and appearance. To rule out this possibility, in Experiment 2, we set up a learning phase to deepen the cognition of the difference between a surgical mask and the N95 mask. The purpose of Experiment 2 was to explore further whether mask type affects the perception of being gazed at. Importantly, by analyzing the 10 excluded data in Experiment 1, it was found that these participants’ CoDGs were too wide and could not be fitted mainly because the proportion of participants judging the gaze direction as averted gaze was too low under the condition of left and right 10°. Therefore, in order to reduce the exclusion rate of participants, we added the conditions that gaze direction was left and right 12°in Experiment 2.

### Methods

#### Participants

Referring to the valid data amount of Experiment 1 and considering that the addition of gaze direction levels (looking at left and right 12° levels were added under gaze direction variable) in Experiment 2 would result in less participant data exclusion, we recruited 31 new participants (10 males, 21 females), aged 18–22 years (*M* = 20.25 *SD* = 1.04), none of whom had participated in Experiment 1. All the participants had normal or corrected-to-normal vision and self-reported absence of mental illness.

#### Stimuli and procedure

The same model pictures used in Experiment 1 were used in Experiment 2 as well, though the pictures of two models (1 male, 1 female) were excluded to accommodate the new design. Similar to Experiment 1, each model displayed a neutral expression which either had 0° (direct gaze), 2°, 4°, 6°, 8°, 10°, or 12° shift of gaze to the right or left (averted gaze).

The entire experiment consisted of two sequential parts: the learning task and CoDG task. In the learning phase, the participants were presented with the knowledge comparison of N95 masks and surgical masks, including three aspects: filtration layer, protection effect and recommended wearing place. 20s later, the participants were presented with the shape of both masks, so that they could be familiar with the shape of different mask types (10s), and finally 5 test questions were presented to test the learning effect of the participants.

The CoDG task procedure was consistent with Experiment 1, except each participant observed three face types for each model. Each participant completed a total of 624 trials (16 trials per face type × gaze direction). The whole experiment lasted about 30 min.

The data analysis was identical to Experiment 1. Four participants were excluded because their response curves were too broad to allow confident estimations of the width of their CoDG, leaving data from 27 participants to be studied in the statistical analysis (7 males, 20 females). The others are the same as Experiment 1.

### Results and discussion

#### Cone of direct gaze

**FIGURE 3:**
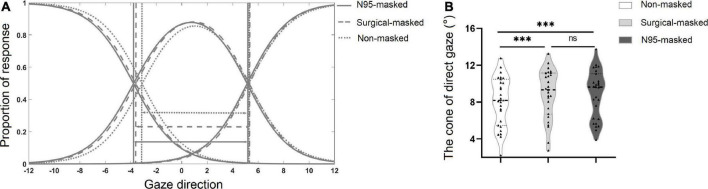
**(A)** Plot showing the mean fitted logistic functions for *left*, *right*, and *direct* responses. The solid lines indicate the N95-masked face condition, the dashed lines indicate the surgical-masked face condition, while the dotted lines indicate the non-masked faces condition. The arrows indicate the width of the cone of direct gaze. **(B)** The mean width of the cone for non-masked face, surgical-masked face, and N95-masked face conditions. ▲ Indicated data for each participant. ^***^p < 0.001, ns *p* > 0.05.

ANOVA test showed a significant main effect of the face type on CoDG, [*F*(2, 52) = 12.74, *p* < 0.001, η*_*p*_*^2^ = 0.329] (see Table 1). A Bonferroni corrected *post hoc* test found that the CoDG for N95-masked faces and surgical-masked faces were higher as compared to the non-masked faces, *p*s < 0.001. However, there was no significant difference between the CoDG for surgical-masked faces and N95-masked faces, *p* > 0.05 (see Figure 3).

#### Correlations between cone of direct gaze and questionnaires

To assess the relationship between the CoDG and the participants’ knowledge about masks as well as between the CoDG and traits of anxiety and depression, we calculated Pearson correlations between the CoDG of a particular face type and the participants’ responses to questions about face masks, SDS, and SIAS. The Bonferroni correction revealed there was no significant correlation, *p*s > 0.05 (see Supplementary Appendix 2).

Experiment 2 replicated the results of Experiment 1 and found that the CoDG for N95-masked faces was significantly wider than that for the non-masked faces, suggesting that wearing masks made individuals judge being looked at more.

## Experiment 3

The results of Experiment 1 and 2 showed that wearing a mask widens the CoDG. One possibility behind this is the significant difference in the social significance of masked and unmasked faces. In social interactions, masked faces are more likely to convey that the mask-wearer is safer to others or the surrounding environment is more threatening as compared to unmasked faces (Tateo, 2021). However, in addition to the difference in social significance, there are differences in the low-level facial features of masked and unmasked faces as well. Unmasked faces have their complete facial features intact and in view while masked faces, due to the physical barrier caused by masks, prevent the viewer from gathering information about the person’s mouth. Previous studies have found that the lower part of the face also conveys a lot of information (Robert and Adam, 2016) and that mask coverings can impact cognitive processing based on information from the part (Carbon, 2020; Noyes et al., 2021). Therefore, there may be another explanation for the results of Experiment 1 and 2. The lack of information related to the mouth obstructs the information processing of masked faces, making it less accurate for individuals to judge eye gaze information and leading them to interpret a more averted gaze as a direct one, thus widening the CoDG. Hence, in Experiment 3, faces with the mouth edited out were employed to address whether the influence of wearing masks on CoDG is a result of the difference in the social significance between masked and non-masked faces or if it is a result of the difference in facial feature information. If the effect of wearing masks on CoDG is derived from the low-level facial feature information, we should observe that the mouth-cut faces have a CoDG that is similar to the N95-masked faces and significantly larger than non-masked faces. If the CoDG of the mouth-cut faces are similar to that of the non-masked faces, and both of their CoDGs are smaller than that of the N95-masked faces, it will indicate that the influence of wearing masks on CoDG is mainly due to the high-level social significance of masked faces.

### Methods

#### Participants

Referring to the valid data amount of Experiment 1 and 2, we recruited 37 new participants (15 males, 22 females), aged 18–30 years (*M* = 20.3 *SD* = 2.05), none of whom had participated in either Experiment 1 or Experiment 2. All the participants had normal or corrected-to-normal vision and self-reported absence of mental illness. The others are the same as Experiment 1.

#### Stimuli and procedure

We created mouth-cut faces by editing out the lower part of the models’ faces (see Figure 1). The same model pictures used in Experiment 2 was used in Experiment 3 as well.

The procedure was consistent with Experiment 1, except that each participant viewed all the faces of one model under three conditions (non-masked face, mouth-cut face, and N95-masked face). Each participant completed a total of 624 trials (16 trials per face type × gaze direction). The whole experiment lasted about 30 min.

The procedure of CoDG task and data analysis were identical to Experiment 1.

The data analysis was identical to Experiment 1. Seven participants were excluded because their response curves were too broad to allow confident estimations of the width of their CoDG, leaving data from 30 participants to be studied in the statistical analysis (10 males, 20 females).

### Results and discussion

#### Cone of direct gaze

**FIGURE 4:**
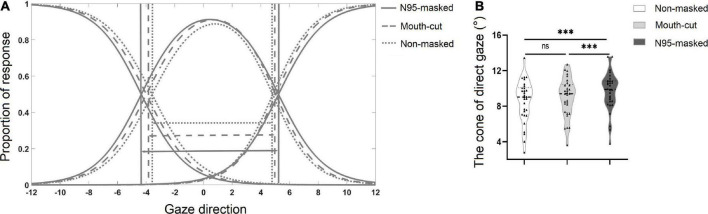
**(A)** Plot showing the mean fitted logistic functions for *left*, *right*, and *direct* responses for N95-masked face, mouth-cut face and non-masked face conditions. The solid lines indicate the N95-masked face condition, the dashed lines indicate the mouth-cut face condition, and the dotted lines indicate non-masked face condition. The arrows indicate the width of the cone of gaze. **(B)** The mean width of the cone for the non-masked, mouth-cut, and N95 -masked conditions. ▲ Indicated data for each participant. ^***^p < 0.001, ns *p* > 0.05.

A one-way repeated-measures ANOVA test showed significant main effect of the condition of the face on the CoDG, [*F*(1.47, 42.58) = 10.61, *p* < 0.001, η*_*p*_^2^* = 0.268] (see Table 1). A Bonferroni corrected *post hoc* test found that the CoDG of N95-masked faces was higher than the mouth-cut faces and non-masked faces, *p*s < 0.001. However, there was no significant difference between the mouth-cut faces and non-masked faces, *p* > 0.05 (see Figure 4).

#### Correlations between cone of direct gaze and questionnaires

To test the relation between the widening of the CoDG and the self-assessment questionnaires, we calculated the Pearson’s correlation coefficients under two conditions, N95-masked minus non-masked and the CoDG obtained when the value of the non-mask faces is subtracted from that of mouth-cut faces (mouth-cut minus non-masked face), drawing on the answers to the COVID-19 questionnaire (Q1, Q2, and Q3), the SIAS score, and the SDS score. Bonferroni correction revealed there was no significant correlation, *p*s > 0.05 (see Supplementary Appendix 2).

To further examine the relationship between the widening CoDG caused by wearing N95 masks and the self-assessment questionnaires, we combined the data from Experiments 1, 2 and 3 under the N95-masked face and non-masked face conditions and calculated the Pearson’s correlation coefficient for N95-masked minus non-masked, drawing on the answers to the COVID-19 questionnaire (Q1, Q2, and Q3), the SIAS score, and the SDS score. There was no significant negative correlation between questionnaires and the widening effect, *ps* > 0.05 (see Supplementary Appendix 2).

Experiment 3 replicated the results of Experiment 1 and 2 and found that the CoDG for N95-masked faces was significantly wider than that for the non-masked faces, suggesting that wearing masks made individuals judge being looked at more. Notably, we found that the CoDG for mouth-cut faces was narrower than that for N95-masked faces while no significant difference was found between the CoDG of mouth-cut faces and non-masked faces. This finding indicated that the influence of wearing masks on the CoDG is due to its high-level social significance rather than the low-level facial feature information.

## General discussion

We conducted a gaze discrimination study to investigate the influence of wearing masks on CoDG. The results showed that wearing masks increases the width of CoDG. We also found that the CoDG of masked faces was significantly wider than that of unmasked faces. This indicated that wearing a mask would affect the processing of gaze direction. However, mouth-cut faces did not produce the same widening effect on CoDG as the masked faces. These findings show that the influence of masks on CoDG can mainly be attributed to the high-level social significance of wearing a mask, rather than the low-level physical information of the face.

Why does wearing a mask influence the judgment of gaze direction? One possible explanation is that wearing face masks increases the sense of trust and, thus, widens the CoDG. During the COVID-19 pandemic, most governments as well as the WHO recommended wearing face masks as a key measure to protect people from the novel coronavirus (Bhardwaj and Agrawal, 2020; Carbon, 2020). In addition, studies have found that, in the context of COVID-19, a face covered by a mask may be considered safer and more trustworthy (Cartaud et al., 2020). The increase in trust makes people willing to interact and, thus, more likely to judge that a face wearing a mask is looking at them. On the contrary, another explanation is that wearing face masks increases the sense of threat and, thus, widens the CoDG. During the COVID-19 pandemic, wearing face masks implies a risk of COVID-19 transmission for the wearer probably being a COVID-19 patient. Consequently, the mask could be perceived as a threat message highly relevant to one’s health. When facing threatening facial expressions or in a threatening situation, previous studies have shown that people tend to judge others as looking at them, resulting in a wider CoDG (Ewbank et al., 2009; Tso et al., 2012; Jun et al., 2013; Harbort et al., 2017). Previous studies have found that masks both increase trust and shorten the social distance, but also increase perceptions of sickness, possibly because the internalized rule of wearing a mask inhibits automatic evaluation of mistrust (Rosa, 2020).

The result of these two contradictions may be that people themselves may hold ambivalent attitudes toward masks. From a cultural psychological perspective, in the context of the COVID-19 pandemic, masks are given some social significance and are no longer neutral objects. Meanwhile, the mask becomes part of the body by covering the nose and mouth areas. It is an interface that simultaneously distances and connects me to the other. By wearing a mask, the person generates different levels of meaning and automatic hetero-regulatory processes (Tateo, 2021). Therefore, we did not specifically discuss whether the widening effect is due to an increased sense of security or threat from masks during the COVID-19 pandemic.

Besides, no significant impact of the mask types on the CoDG emerged; as the same widening effect on the CoDG was observed for both the N95-masked faces and surgical-masked faces. On the one hand, during the pandemic, people have been highly sensitive to the threatening information about COVID-19. As for information about the level of threat, masks that offer lower protection (surgical-masked) and those that offer higher protection (N95-masked) can induce the alert response from individuals. Therefore, the N95-masked and surgical-masked faces have the same widening effect. Apparently, such an undifferentiated alert response has adaptive significance for human survival. To give an example, although there was a difference in the feasibility of transmitting the virus between COVID-19 patients and those who had recovered, there was no difference in viewing time to their faces (Federico et al., 2021). In general, individuals have the same avoidance response to novel coronavirus-related information at different threat levels.

On the other hand, there is no significant difference in the cognition of mask protection in the individual’s perception. This may have something to do with advice circulated by the media to the general public to wear face masks during the pandemic, that both surgical masks and N95 masks provide adequate protection against novel coronavirus transmission through droplets (Sureka et al., 2020). Furthermore, people are equally familiar with and are likely to be exposed to both types of masks. Consequently, this may result in the inability to observe the different effects of mask types in our study. Future studies may use more diverse mask types having different protective characteristics (such as gauze masks vs. N95 masks) to further explore this issue.

The present study did not find any correlation between the three-item questionnaire on COVID-19 and scale and the mask effect, which was consistent with the results of previous studies (Lobmaier et al., 2021). The correlation results might be related to the sample size, and future studies could use larger samples to verify the correlation between individual traits and the mask effect. Another possible reason was that our participants were selected from college students and did not include clinically diagnosed patients, so masking the correlation between the questionnaire and CoDG. This could be further explored in the future by selecting clinical participants.

Another key point to remember is that there were some limitations in the participant selection process, since all of them were recruited from college. Inevitably, age and knowledge of background may have influenced their gaze judgments. Considering this, it would be useful for future studies to recruit people of different ages and backgrounds. It must also be noted that the participants were all Chinese. Previous studies have reported the significance of the culture of face masks (Timpka and Nyce, 2021) and different cultural effects on the perception of facial information (Blais et al., 2008; Jack et al., 2009). Meanwhile, during the COVID-pandemic, different cultures took different measures and also showed different attitudes to wearing masks. As such, further studies are required to compare the effects of face masks on gaze perceptions in different cultures. In addition, considering that taking physical masking approaches would introduce new variables (e.g., color, personal preference, etc.), the current study created mouth-cut faces by editing out the lower part of the models’ faces as the control condition. However, this operation could disrupt the integrity of a face. Previous studies found the presentation of a whole face affects the processing of face information (Leder and Carbon, 2005). Thus, further studies could seek out a better control condition to explore the widening effect of face masked on CoDG.

Furthermore, with the global outbreak of COVID-19 pandemic and the virus mutating, people have become fully aware of the seriousness of the epidemic and the wearing of masks has become widely recognized during the period of data collection (March 2021 to December 2021). Many countries have now announced the removal of epidemic prevention and control measures, and it is possible that people’s perceptions of masks may vary, so the results may not apply to those who are not required to wear them.

## Conclusion

The current study adapted a gaze direction judgment methodology to measure the influence of masks on CoDG. The results provide novel evidence linking the wearing of masks to the widening of an individual’s CoDG. Furthermore, the widening effect may be related to the social meaning induced by face mask, rather than physical barrier. The frequency with which individuals wear masks during the COVID-19 pandemic may reduce the influence of masks on the CoDG. In addition, it was found that the mask type does not regulate the effect of the mask on the CoDG. This study has significant implications for understanding the impact of wearing masks on human social perceptions in the backdrop of COVID-19.

## Data availability statement

The raw data supporting the conclusions of this article will be made available by the authors, without undue reservation.

## Ethics statement

The studies involving human participants were reviewed and approved by the Ethics Committee of the Institute of Brain and Psychological Sciences, Sichuan Normal University. The participants provided their written informed consent to participate in this study. Written informed consent was obtained from the model for the publication of her images in this article.

## Author contributions

ZH conceived and designed the experiments. JL, LH, LZ, and JX performed the data acquisition and analyzed the data. JL, ZH, and JY interpreted the data and drafted the manuscript. All authors revised and approved the manuscript.
