# Supplementary material for: Masked face is looking at me: Face mask increases the feeling of being looked at during the COVID-19 pandemic
Source: Front Neurosci. 2022 Nov 24;16:1056793. doi: 10.3389/fnins.2022.1056793 (PMC9730803; doi:10.3389/fnins.2022.1056793)
Supplement: Supplementary file 1 [file Data_Sheet_1.docx]

Appendix 1

***Supplementary details of participants***

This study recruited participants on university campuses by placing promotional advertisements. All participants received monetary compensation. Participants were recruited inside the university campus. And all of them self-reported that they had not participated in similar experiments.

***Supplementary details of Procedure***

Each participant was taken to the same behavioral laboratory upon arrival to ensure no additional variables were created. They were then informed of the entire experiment's flow, and signed informed consent. Next, they completed a gaze direction judgment task (the CoDG task), followed by completing questionnaires.

***The instruction for the CoDG task in Experiments***

In the following experiment, a picture of a face is first presented in the center of the screen. Your task is to determine in which direction the eyes of the person in the picture are looking. If you feel like looking to the left, press 1; if you feel like looking to the right, press 3; if you feel like looking at you, press 2. Please use your first sense to make a judgment. After understanding the experiment task, please press any key to start the experiment.

***Details for Learning phase in Experiment 2***

The knowledge of the Surgical mask vs N95 mask (20s)

| Mask type | Surgical mask | N95 mask |
| --- | --- | --- |
| Number of filter layers | ≥3 | 3 |
| Protection effect | 98.1% | 38.8% |
| Recommended places of use | Higher risk | Low risk |

Mask form (10s)


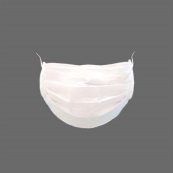

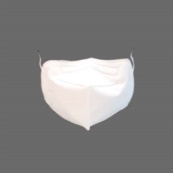


Surgical mask N95 mask

Five items of test:

Q1: Which mask should be worn by health care workers in fever clinics?

1.Surgical mask

2.N95 mask

Q2: Which mask has better protection?

1.Surgical mask

2.N95 mask

Q3: Which mask recommend to wear in low-risk areas?

1.Surgical mask

2.N95 mask

Q4: Which mask is surgical mask?

1.
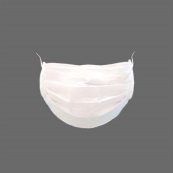


2.
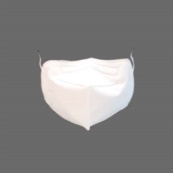


Q5: Which mask is N95 mask?

1.
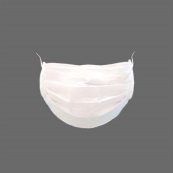


2.
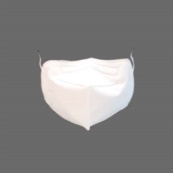


The order of five questions was random.
