# Supplementary material for: Masked face is looking at me: Face mask increases the feeling of being looked at during the COVID-19 pandemic
Source: Front Neurosci. 2022 Nov 24;16:1056793. doi: 10.3389/fnins.2022.1056793 (PMC9730803; doi:10.3389/fnins.2022.1056793)
Supplement: Supplementary file 2 [file Data_Sheet_2.docx]

Appendix 2

Table S1

*Pearson correlation of the difference between CoDG and self-assessment questionnaire scores in Experiment 1, 2, and 3*

|  | Experiment 1 | | Experiment 2 | | Experiments 3 | | | Experiments 1, 2 & 3 |
| --- | --- | --- | --- | --- | --- | --- | --- | --- |
|  | Surgical-masked minus non-masked | N95-masked minus non-masked | Surgical-masked minus non-masked | N95-masked  minus non-masked | | N95-masked  minus non-masked | Cut-mouth minus non-masked | N95-masked  minus non-masked |
| Q1^a^ | -0.41 | -0.28 | 0.18 | 0.07 | -0.23 | | -0.04 | -0.17 |
| Q2^b^ | -0.12 | -0.03 | 0.45 | -0.29 | 0.30 | | 0.22 | 0.04 |
| Q3^c^ | -0.31 | -0.07 | 0.04 | -0.3 | -0.09 | | 0.07 | -0.10 |
| SIAS^d^ | 0.13 | 0.44 | 0.14 | -0.24 | -0.23 | | -0.11 | -0.05 |
| SDS^f^ | -0.03 | -0.33 | 0.13 | -0.04 | 0.10 | | 0.04 | 0.05 |

Note:

* Correlation is significant at the 0.05 level (2-tailed).

(a) Q1 = I wear a mask when I go out during the COVID-19 pandemic;

(b) Q2 = I think masks played a big role in protection during the COVID-19 pandemic;

(c) Q3 = In public, I prefer to communicate with people wearing masks;

(d) SIAS = Social Interaction Anxiety Scale;

(f) SDS = Self-Rating Depression Scale.
